# Impact of cigarette tax increase on health and financing outcomes in four Indian states

**DOI:** 10.12688/gatesopenres.13127.1

**Published:** 2020-05-11

**Authors:** Daphne C. Wu, Vikas Sheel, Pooja Gupta, Beverley M. Essue, Linh Luong, Prabhat Jha

**Affiliations:** 1 Centre for Global Health Research, St. Michael's Hospital, Toronto, Toronto, Ontario, M5B 1W8, Canada; 2 Ministry of Health and Family Welfare, Government of India, New Delhi, New Delhi, India

**Keywords:** tobacco tax, extended cost-effectiveness analysis, tobacco economics, subnational, India

## Abstract

**Background**: In India, about one million deaths occur every year due to smoking. Tobacco taxation is the most effective intervention in reducing smoking. In this paper, we examine the impact of a one-time large cigarette price increase, through an increase in excise tax, on health and financing outcomes in four Indian states.

**Methods**: We used extended cost-effectiveness analysis to estimate, across income quintiles, the life-years gained, treatment cost averted, number of men avoiding catastrophic health expenditures and extreme poverty, additional tax revenue collected, and savings to the Ayushman Bharat Pradhan Mantri Jan Arogya Yojana (AB-PMJAY) with a cigarette price increase to Indian Rupees (INR) 10 plus 10% *ad valorem* in four Indian states.

**Results**: With the price increase, about 1.5 million men would quit smoking across the four states, with the bottom income group having 7.4 times as many quitters as the top income group (485,725 vs 65,762). As a result of quitting, about 665,000 deaths would be averted. This would yield about 11.9 million life-years, with the bottom income group gaining 7.3 times more than the top income group. Of the INR 1,729 crore in treatment cost averted, the bottom income group would avert 7.4 times more than the top income group. About 454,000 men would avoid catastrophic health expenditures and 75,000 men would avoid falling into extreme poverty. The treatment cost and impoverishment averted would save about INR 672 crore in AB-PMJAY. The tax increase would in turn, generate an additional tax revenue of about INR 4,385 crore. In contrast to the distribution of health benefits, the extra revenue generated from the top income group would be about 3.1 times that from the bottom income group.

**Conclusions**: Cigarette tax increase can provide significant health and economic gains and is a pro-poor policy for India.

## Introduction

Tobacco use is the leading cause of preventable premature mortality globally ^1^. In India, about one million deaths occur every year due to tobacco smoking ^2^. Cessation substantially reduces the risk of dying; in particular cessation before age 40 reduces the risk of death associated with continued smoking by about 90% ^3^. Yet, cessation remains uncommon in India. According to the Global Adult Tobacco Survey (GATS), in 2009–10, 5.7% of adults aged 15 years and above (10.3% males, 0.8% females) smoke cigarettes in India ^4^; this only decreased to 4.0% (7.3% males, 0.6% females) in 2016–17 ^5^. Tobacco use is also more prevalent among the poor, who in turn, fall into greater poverty, as poor families spend a larger proportion of their income on tobacco and are at a much higher risk of falling ill and dying from smoking-attributable diseases, thus imposing additional costs to the family ^6^.

The most effective intervention to reduce tobacco use is a large increase in the federal excise tax on cigarettes that enables minimal downward substitutions to shorter, cheaper cigarettes ^1, 7– 9^. Evidence from several low- and middle-income countries (LMICs) has shown that the impact of tobacco taxation would disproportionately favour populations on lower income ^10– 15^. To date, however, no study has examined the impact of tobacco tax increase across income groups at the subnational level. In this study, to support tobacco control policies through increased tobacco taxation in India, we estimated the impact of a cigarette price increase through an increase in excise tax from the 2018-19 tax structure (Underlying data ^16^) to 10 Indian Rupees (INR) plus 10% *ad valorem*, across income groups in four states in India.

## Methods

We used the extended cost-effectiveness analysis (ECEA) model that was developed in the Disease Control Priorities Project ^17^, and was previously used to estimate the impact of a 50% increase in the price of cigarettes on health, poverty, and financial outcomes in 13 middle-income countries, and the impact of cigarette price increase in Vietnam ^12, 14^. The model was used to estimate the impact across five income groups in four states in India of a cigarette price increase, resulting from a tax increase from the 2018-19 tax structure to INR 10 plus 10% *ad valorem* for all lengths of filtered cigarettes on smoking reduction, deaths averted due to major tobacco-attributable diseases (chronic obstructive respiratory disease (COPD), stroke, heart disease and cancer), life-years gained, treatment cost averted, number of men avoiding catastrophic health expenditures and extreme poverty, additional tax revenues raised from the tax increase, and cost savings to the Ayushman Bharat Pradhan Mantri Jan Arogya Yojana (AB-PMJAY).

### Study population

We selected four states in India, namely Karnataka, Assam, Uttar Pradesh, and Maharashtra, based on recommendations from the Indian Ministry of Health and Family Welfare. These four states are also characterized by diverse socioeconomic demographic characteristics, tobacco use, and health insurance coverage. We focussed on male smokers aged 15 years and older, as they constitute more than 90% of all cigarette smokers in India ^5^. To estimate the number of smokers by age and income groups in each state, we applied the age-specific smoking prevalence for males in each state from the second round of GATS survey in India in 2016–17 to the male population in each state ^4^. The male population in each age group for each state was estimated demographic data from the 2011 Census of India, applied to the Indian male population and projected by the United Nations Population Division for 2018 ^18, 19^. As the GATS survey did not report prevalence based on income, we used education level as a proxy for income group, such that those with no formal education were classified as the poorest 20% of the population and those with college education or higher were classified as the richest 20% of the population.

### Cigarette price and price increase

We calculated the base price of the different lengths of filtered cigarettes sold in India (less than 65 mm, 65–70 mm, 70–75 mm and longer than 75mm) using data from Market price A.C. Nielson for 2014 ^20^, and inflated the price using the inflation rate in India for 2018 obtained from the World Bank Development Indicators ^21^. We focussed only on filtered cigarettes as they constitute more than 92% of the market shares in India. Applying the Goods and Services Tax (GST), GST Compensation Cess and the National Calamity Contingency Duty (NCCD) in accordance with the tax structure for cigarettes in India in 2018-19 to the base price ^22^, we obtained the 2018-19 market price of a pack of 20 cigarettes. Using the proposed tax of INR 10 (which includes GST, GST Compensation Cess, and NCCD) plus 10% *ad valorem*, we calculated the percentage increase in market price. We assumed that the tax increase will be passed on to consumer prices, as recent analyses in India showed that in nearly all cases, most of the tax hikes were passed onto smokers, but tax decreases did not reduce consumer prices ^23^. The market price was assumed to be uniform in all of the four states studied.

### Price elasticity

For this study, we considered a conservative scenario and used the average price elasticity for cigarette demand in both high and low- and middle-income countries of -0.40 ^12, 24, 25^. The price elasticity in India is likely to be higher, as John (2008) reported a price elasticity of -0.34 ^26^. We used this elasticity in our sensitivity analysis (see Sensitivity Analysis section below). Research suggests that young people and smokers who are on low income are more price sensitive than older people and smokers who are on high income ^24, 27^. Hence, we used two times the price elasticity (-0.80) for young smokers aged 15–24 years, as reported by the U.S. Department of Health and Human Services and International Research Agency for Cancer ^24, 28^. For smokers in the lowest income quintile and those in the highest income quintile, we used the price elasticity of -0.64 and -0.12, as used by the Global Tobacco Economics Consortium (GTEC) ^12^. We also assumed that the price elasticity of quitting is half of the price elasticity of demand.

### Effects of cigarette price increase on quitting, life-years gained, disease costs, income poverty, tax revenue, and cost savings to AB-PMJAY

We used the ECEA model to estimate the impact of the cigarette price increase on quitting, number of deaths attributable to four major tobacco-attributable diseases (chronic obstructive respiratory disease (COPD), stroke, heart disease and cancer) averted, life-years gained, treatment cost averted (all treatment cost, most of which are paid out of pocket in India) due to the four tobacco-attributable diseases, number of men avoiding catastrophic health expenditures and extreme poverty, as defined by the World Bank as income of under $1.90 per day in purchasing power parity, and additional tax revenues collected ^12, 14^. To estimate the cost savings to AB-PMJAY─ the National Health Protection Scheme in India which provides a health insurance cover of INR 5 lakh to families living below the poverty line─ we added the cost savings in AB-PMJAY due to men prevented from falling below the poverty line to the treatment cost for those living below the poverty line before the tax increase. To obtain the former cost, we applied a 50% risk of dying from tobacco-attributable diseases, the proportion of smoking-related deaths due to COPD, stroke, heart disease, and cancer obtained from the Indian Million Death Study ^2^, and the treatment cost of each disease to the number of men who would otherwise have continued to smoke and hence, fall below the poverty line. The data inputs and sources of data are available as *Underlying data* ^16^. For indicators where state-level estimates could not be obtained, national estimates were used. All treatment costs obtained from the literature were converted to INR and inflated to the costs in 2018 using the exchange rate and consumer price index obtained from the World Bank Development Indicators ^21^.

### Sensitivity analysis

We conducted sensitivity analyses to examine the impact of a 25% and 100% price increase with the price elasticity of demand for cigarettes of -0.40, and that of INR 10 plus 10% ad valorem with the cigarette price elasticity of -0.34 in India ^26^. For the lowest income group, we used a price elasticity of -0.635, as done by GTEC ^12^.

All analyses were performed using Stata version 15.1 ^29^.

## Results

### Smoking prevalence in Karnataka, Assam, Uttar Pradesh and Assam

Among the four states studied, Assam has the highest male cigarette smoking prevalence of 9.7% ( Table 1), and Uttar Pradesh has the highest absolute number of cigarette smokers of about 6.5 million men. Maharashtra has the lowest smoking prevalence overall as well as for almost all age groups. In Assam and Maharashtra, cigarette smoking prevalence is higher among the higher income groups, whereas in Uttar Pradesh, the prevalence is higher among the lower income groups. In Karnataka, the prevalence was comparable across all income groups.

**Table 1.** Prevalence of cigarette smoking in 4 states in India: Karnataka, Assam, Uttar Pradesh, and Maharashtra, by age and income groups.

	Karnataka	Assam	Uttar Pradesh	Maharashtra
**Overall**	6.6	9.7	8.6	2.7
**Age groups**				
15–29	7.0	12.9	7.1	2.9
30–44	7.0	7.6	13.2	3.4
45–59	6.5	9.3	6.7	1.7
60–69	4.7	2.5	6.6	3.0
≥70	5.2	5.9	4.3	0.5
**Income groups**				
First (bottom 20%)	7.3	4.8	11.4	2.8
Second	7.3	7.8	20.9	1.2
Third	7.6	11.1	8.0	2.6
Fourth	4.5	11.6	6.3	2.6
Fifth (top 20%)	7.8	12.7	3.2	4.8

Before the price increase, an estimated total of about 10.7 million males older than 15 years smoked cigarettes across all of the four states studied ( Table 2). Men in the bottom income group (poorest 20% of the population) accounted for 21%, while men in the top income group (richest 20% of the population) accounted for 15% of the total number of male smokers.

**Table 2.** Cumulative impact of a cigarette tax increase to INR 10 plus 10 % *ad valorem* on health and financing outcomes in 4 states in India: Karnataka, Assam, Uttar Pradesh, and Maharashtra.

Variables by income groups	Karnataka	Assam	Uttar Pradesh	Maharashtra	Four states total
**Number of male smokers aged ≥15 years before price increase (in thousands)**					
First (bottom 20%)	364.1	119.8	1,492.2	270.4	2,246.5
Second	361.8	193.2	2,727.5	114.0	3,396.5
Third	373.3	273.1	1,041.3	250.1	1,937.7
Fourth	219.9	286.3	816.3	255.1	1,577.7
Fifth (top 20%)	385.0	314.8	422.9	465.6	1,588.4
Total	1,704.2	1,187.1	6,500.2	1,355.3	10,746.8
First: fifth ratio	0.9	0.4	3.5	0.6	1.4
**Number of males who quit smoking due to intervention (in thousands)**					
First (bottom 20%)	75.7	31.9	322.7	55.4	485.7
Second	60.0	41.0	470.0	18.6	589.6
Third	46.1	43.1	133.7	30.4	253.4
Fourth	17.9	29.8	69.0	20.4	137.0
Fifth (top 20%)	15.0	15.7	17.2	17.9	65.8
Total	214.7	161.4	1,012.6	142.8	1,531.5
First: fifth ratio	5.0	2.0	18.8	3.1	7.4
**Total deaths averted due to COPD, stroke, heart disease, and cancer (in thousands)**					
First (bottom 20%)	32.3	14.5	139.8	23.6	210.3
Second	25.6	18.6	203.7	7.9	255.8
Third	19.7	19.6	57.9	13.0	110.2
Fourth	7.6	13.5	29.9	8.7	59.7
Fifth (top 20%)	6.4	7.1	7.4	7.6	28.6
Total	91.7	73.3	438.7	60.9	664.7
First: fifth ratio	5.0	2.0	18.8	3.1	7.4
**Total life-years gained (in thousands)**					
First (bottom 20%)	574.7	265.5	2,489.9	421.8	3,751.9
Second	455.1	341.3	3,626.6	141.7	4,564.7
Third	349.8	359.5	1,031.6	231.7	1,972.6
Fourth	135.6	248.0	532.0	155.5	1,071.1
Fifth (top 20%)	113.9	130.9	132.3	136.2	513.3
Total	1,629.1	1,345.1	7,812.5	1,086.9	11,873.7
First: fifth ratio	5.0	2.0	18.8	3.1	7.3
**Treatment cost averted (in INR, crores ($Int, millions))**					
First (bottom 20%)	84.4 (46.6)	36.6 (20.2)	289.0 (159.6)	57.4 (31.7)	467.4
Second	103.0 (56.9)	43.7 (24.1)	593.0 (327.5)	22.9 (12.6)	762.6
Third	50.3 (27.8)	50.1 (27.7)	148.0 (81.7)	33.2 (18.3)	281.6
Fourth	38.8 (21.4)	28.3 (15.6)	63.4 (35.0)	23.6 (13.0)	154.1
Fifth (top 20%)	17.7 (9.8)	13.3 (7.3)	18.8 (10.4)	13.7 (7.6)	63.5
Total	294.2 (162.5)	172.0 (95.0)	1,112.2 (614.3)	150.8 (83.3)	1,729.2
First: fifth ratio	4.8	2.8	15.4	4.2	7.4
**Number of men avoiding catastrophic health expenditures (in thousands)**					
First (bottom 20%)	22.6	9.8	77.7	15.4	125.6
Second	27.2	11.7	159.3	6.1	204.4
Third	10.6	13.4	39.7	7.4	71.2
Fourth	8.1	7.6	17.0	5.0	37.7
Fifth (top 20%)	3.4	3.6	5.0	2.8	14.7
Total	71.9	46.2	298.8	36.7	453.6
First: fifth ratio	6.7	2.8	15.4	5.6	8.5
**Number of men avoiding extreme poverty (in thousands)**					
First (bottom 20%)	0.0	1.6	40.1	0.0	41.8
Second	0.0	1.1	25.1	0.0	26.2
Third	0.0	0.0	6.2	0.0	6.3
Fourth	0.0	0.0	0.9	0.0	0.9
Fifth (top 20%)	0.0	0.0	0.0	0.0	0.0
Total	0.0	2.6	72.3	0.0	75.1
First: fifth ratio	-	-	>100	-	>100
**Cost savings to AB-PMJAY (in INR, crores ($Int, millions))**					
Total	0.1 (0.0)	54.7 (30.2)	617.7 (341.2)	0.1 (0.0)	672.5
**Additional tax revenues (in INR, crores ($Int, millions))**					
First (bottom 20%)	227.9 (125.9)	35.8 (19.8)	96.5 (53.3)	99.6 (55.0)	459.9 (254.0)
Second	122.0 (67.4)	61.9 (34.2)	231.5 (127.9)	33.4 (18.4)	448.8 (247.9)
Third	349.5 (193.1)	87.6 (48.4)	239.0 (132.0)	179.2 (99.0)	855.3 (472.4)
Fourth	336.2 (185.7)	212.8 (117.5)	415.0 (229.2)	216.8 (119.8)	1,180.7 (652.2)
Fifth (top 20%)	397.5 (219.5)	240.2 (132.7)	483.4 (267.0)	319.3 (176.3)	1,440.3 (795.5)
Total	1,433.1 (791.5)	638.2 (352.5)	1,465.4 (809.4)	848.2 (468.5)	4,385.0 (2,422.0)
First: fifth ratio	0.6	0.1	0.2	0.3	0.3

### Impact of tax increase to INR 10 + 10% ad valorem across Karnataka, Assam, Uttar Pradesh and Assam

In 2018-19, the average market price of filtered cigarettes for a pack of 20 is about INR 224.49. With a tax increase to INR 10 plus 10% *ad valorem*, the price would increase to INR 344.04. This represents a 53.25% price increase. This increase in cigarette price would lead to about 1.5 million men quitting smoking across the four states, with the bottom income group having 7.4 times as many quitters as the top income group (485,725 vs 65,762). An estimated total of 665 thousand deaths due to COPD, stroke, heart disease, and cancer would be averted among current smokers due to quitting. The number of averted deaths in the bottom income group would be 7.4 times that in the top income group (210,289 vs 28,610). The deaths averted due to quitting would yield an estimated 11.9 million life-years, with the bottom income group gaining 7.3 times more life-years than those in the top income group (3,751,930 vs 513,319).

The cost averted for treating the four major tobacco-attributable diseases would amount to more than INR 1,729 crore ($Int 955 million). The treatment cost averted in the bottom income group would be 7.4 times higher than in the top income group (INR 467 crore vs 64 crore, or $Int 258 million vs 35 million). As a result of the total treatment cost averted, about 454 thousand men would avoid catastrophic health expenditures and about 75 thousand men would avoid falling into extreme poverty. The treatment cost and impoverishment averted would save about INR 672 crore ($Int 371 million) in AB-PMJAY. The increase in excise tax would generate an additional tax revenue of about INR 4,385 crore ($Int 2,422 million). In contrast to the distribution of health benefits, the extra revenue generated from men in the top income group would be about 3.1 times that from the bottom income group (INR 1,440 crore vs 460 crore, or $Int 759 million vs 254 million).

### Karnataka

In Karnataka, there are about 1.7 million male cigarette smokers aged 15 and above in 2016–17. Men in the bottom and the top income groups each account for about 21% of the total number of smokers. Smoking prevalence declined modestly with age in those aged 15–29 to 60–69 but increased in those aged 70 and above.

With a 53.25% increase in cigarette price through the excise tax increase, about 215 thousand men would quit smoking, with the bottom income group having five times as many quitters as the top income group (75,743 vs 15,017). Quitting as a result of the price increase would avert about 92 thousand deaths due to COPD, stroke, heart disease, and cancer among male smokers. The number of deaths averted in the bottom income group would be five times that in the top income group (32,353 vs 6,414). As a result of the deaths averted, Karnataka would gain about 1.6 million life-years and avert about INR 294 crore ($Int 162 million) in treatment cost for treating the four tobacco-attributable diseases. The averted treatment cost in the bottom income group would be 4.8 times that in the top income group (INR 84 crore vs 18 crore, $Int 47 million vs 10 million). About 72 thousand men would avoid catastrophic health expenditures, with the bottom income group avoiding 6.7 times that of the top income group (22,618 vs 3,355). The tax increase would generate about INR 1,433 crore ($Int 792 million), of which the top income group would contribute 1.7 times that from bottom income group (INR 297 crore vs 228 crore, $Int 220 million vs 126 million).

### Assam

Of the four states studied, Assam has the highest male cigarette smoking prevalence of 9.7% or about 1.2 million male cigarette smokers. Cigarette smoking prevalence vary considerably across age groups, with the highest prevalence among males aged 15–29 and lowest among males aged 60–69. The prevalence increases with income.

With a 53.25% increase in cigarette price, about 161 thousand men would quit smoking, with the bottom income group having twice as many quitters as the top income group (31,866 vs 15,706). Quitting as a result of the price increase would avert about 73 thousand deaths due to COPD, stroke, heart disease, and cancer among male smokes. The number of deaths averted in the bottom income group would be twice that in the top income group (14,467 vs 7,131). As a result of the deaths averted, Assam would gain about 1.3 million life-years and avert about INR 172 crore ($Int 95 million) in treatment cost for treating the four tobacco-attributable diseases. The averted treatment cost in the bottom income group would be 2.8 times that in the top income group (INR 37 crore vs 13 crore, $Int 20 million vs 7 million). As a result of the treatment cost averted, about 46 thousand men would avoid catastrophic health expenditures, and about 2,635 men would avoid falling into extreme poverty. The treatment cost and impoverishment averted would save about INR 55 crore ($Int 30 million) in AB-PMJAY. The tax increase would generate more than INR 638 crore ($Int 352 million), of which the top income group would contribute 6.7 times that from bottom income group (INR 240 crores vs 36 crore, $Int 132 million vs 20 million).

### Uttar Pradesh

Uttar Pradesh has an overall male cigarette smoking prevalence of 8.6%, but has the highest number of male cigarette smokers among the four study states of about 6.5 million. The prevalence is highest among men aged 30–44 and declines with age to 4.3% among those aged 70 and above. In contrast to Assam, smoking prevalence decreases with income.

With a 53.25% increase in cigarette price, more than 1 million men would quit smoking, with the bottom income group having 18.8 times as many quitters as the top income group (322,707 vs 17,150). Quitting as a result of the price increase would avert about 439 thousand deaths due to COPD, stroke, heart disease, and cancer among male smokers. The number of deaths averted in the bottom income group would be 18.8 times that in the top income group (139,821 vs 7,431). As a result of the deaths averted, Uttar Pradesh would gain about 7.8 million life-years and avert about INR 1,112 crore ($Int 614 million) in treatment cost for treating the four tobacco-attributable diseases. The averted treatment cost in the bottom income group would be 15.4 times that in the top income group (INR 289 crore vs 19 crore, $Int 159 million vs 10 million). Due to the treatment cost averted, about 299 thousand men would avoid catastrophic health expenditure, 72 thousand men would avoid falling into extreme poverty, and about INR 618 crore ($Int 341 million) would be saved in AM-PMJAY. The tax increase would generate about INR 1,465 crore ($Int 809 million), of which the top income group would contribute 5 times that from bottom income group (INR 483 crore vs 96 crore, $Int 267 million vs 53 million).

### Maharashtra

Among the four study states, Maharashtra has the lowest male cigarette smoking prevalence of 2.7%. Smoking prevalence varies across age groups with the highest prevalence among men 30–44 years (3.4%) and lowest among men aged 70 and above (0.47%). Similar to Assam and in contrast to Uttar Pradesh, the prevalence increases with income.

With a 53.25% increase in cigarette price, about 143 thousand men would quit smoking, with the bottom income group having more than three times as many quitters as the top income group (55,410 vs 17,889). Quitting as a result of the price increase would avert about 61 thousand deaths due to COPD, stroke, heart disease, and cancer among male smokers. The number of deaths averted in the bottom income group would be 3.1 times that in the top income group (23,647 vs 7,635). As a result of the deaths averted, Maharashtra would gain about 1 million life-years and avert about INR 151 crore ($Int 83 million) in treatment cost for treating the four tobacco-attributable diseases. The averted treatment cost in the bottom income group would be about 4.2 times that in the top income group (INR 57 crore vs 14 crore, $Int 32 million vs 8 million). About 37 thousand men would avoid catastrophic health expenditures, with the bottom income group avoiding more than five times that of the top income group (15,409 vs 2,775). The tax increase would generate about INR 848 crore ($Int 468 million), of which the top income group would contribute 3.2 times that from bottom income group (INR 319 crore vs 100 crore, $Int 176 million vs 55 million).

### Sensitivity analysis

**Figure 1.**
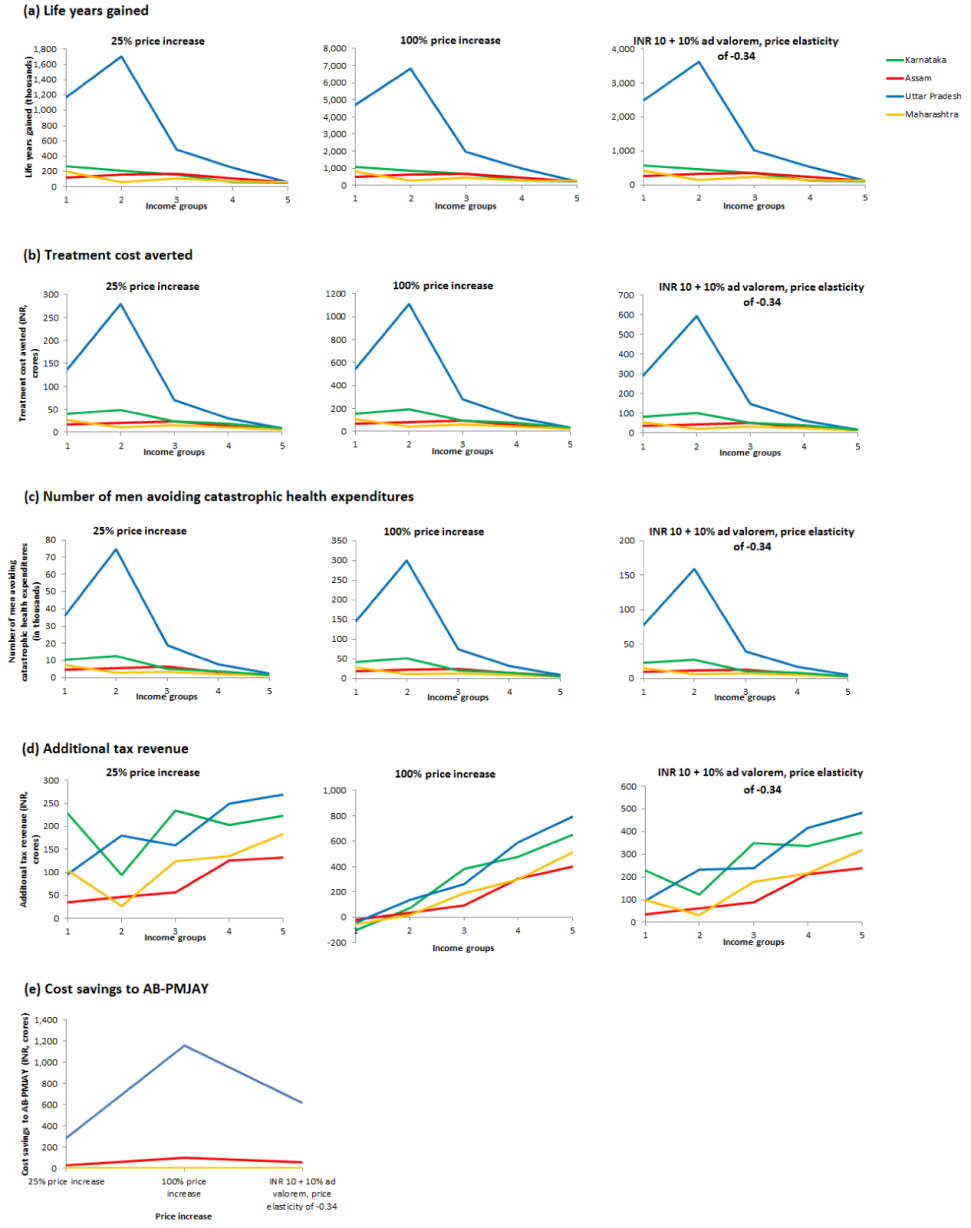
Sensitivity analysis for health and financial outcomes by varying degree of price increase and price elasticity.

Our sensitivity analysis yielded similar results. Across all three scenarios (25% price increase, 100% price increase, and price increase to INR 10 plus 10% *ad valorem* with a price elasticity of -0.34), the ratio of the additional life-years gained, treatment cost averted, and number of men avoiding catastrophic health expenditure between the bottom and the top income groups is similar to that in the baseline scenario ( Figure 1a–c). Compared to the baseline scenario, with a 25% cigarette price increase, the ratio of the additional tax revenue collected from the top income group to the bottom income group increases in all four states, while with a 100% price increase, the bottom income group would accrue tax savings as a result of quitting and reduced consumption ( Figure 1d). The cost savings to AB-PMJAY, in Assam and Uttar Pradesh, from a 25% price increase would be less than half that from the baseline scenario (INR 26 crore 55 crore in Assam, and INR 290 crore vs 618 crore in Uttar Pradesh), while that from a 100% price increase would be almost double (INR 103 crore in Assam and INR 1,160 crore in Uttar Pradesh) ( Figure 1e). The findings using the Indian price elasticity of -0.34 are similar to that of the baseline scenario.

## Discussion

This study confirms that significant tax increases on filtered tobacco would be associated with pro-poor health outcomes as well as significant reductions in poverty and levels of catastrophic health expenditure in the Indian population. Increasing the market price of filtered tobacco is estimated to have a higher impact on the smoking rates of lower income Indian households, and as a result, poorer households bare a lower share of the overall net increased market cost. This study reaffirms that tobacco taxation is an important public policy approach that is associated with a pro-poor impact in India, as also found in other LMICs ^10– 15^.

The findings from this subnational analysis of four states are broadly consistent with the trends seen in the national results ^12^. However, the magnitude of effects differs across states. For example, the ratio between the outcomes between the first and fifth income groups is largest in Uttar Pradesh due to a wide gap in the rates of cigarette smoking between the first and fifth income groups (11.4 vs 6.3%). In this state, the poor would benefit the most from large tax increases and this benefit is significantly higher than the national estimate. The subnational results provide an important economic platform to engage with state-level decision makers and other stakeholders to demonstrate the differential impact that higher cigarette taxes can achieve within their jurisdictions. These results should be used to stimulate action from the local level and foster a groundswell of political buy-in to advance the national agenda to adopt higher tobacco taxes. Leveraging sub-national data to develop an economic case for stronger tobacco taxation policies is a strategy that is currently being pursued in several other settings including Mexico and Colombia ^30^.

The number of men avoiding impoverishment is minimal in Karnataka and Maharashtra, which is likely because of a higher income level overall in these two states. Similarly, cost-savings to AB-PMJAY is also small in these states as fewer people are eligible to AB-PMJAY. While the potential to alleviate poverty was minimal, the higher taxes would be associated with an avoidance of a significant amount of treatment costs and reductions in catastrophic health expenditures in both states and the highest share of these reductions would be among the poorest groups. By applying the ECEA method, we have demonstrated the impact that higher cigarette taxes will have on the two key indicators that are used to assess progress towards achieving financial risk protection and universal health coverage, namely avoidance of illness-related poverty and catastrophic health expenditures ^31^. This work highlights the criticality of this fiscal policy measure for achieving universal health coverage and the Sustainable Development Goals (SDGs).

There is now robust evidence generated from several LMICs that support the role of tobacco taxation policies in achieving significant health outcomes, particularly among the poor, as well as an overall reduction in poverty ^10– 15^. By curbing rates of tobacco-related non-communicable diseases in LMICs, tobacco taxation is also a means to advance economic development in these settings. Yet, the momentum for implementing and sustaining this fiscal policy approach has yet to be achieved in India and other settings. Advancing more aggressive taxation of tobacco across India and other LMICs is urgently needed to align with the recommendations of the FCTC. Recent reports suggest that for most countries, the increases that have been made to date are still far too small to significantly alter smoking rates ^32^.

This study has limitations. First, we used education as the proxy for income. This may underestimate the true socioeconomic status of households. Second, a subset of subnational inputs was not available and so national inputs were used. In some cases this could under or overestimate the effects, though we anticipate the impact would be minimal as national data were substituted for less than 20% of overall inputs. Third, AB-PMJAY has expanded to beyond covering only those living below the poverty line ^33^. Hence, savings to AB-PMJAY due to averted treatment costs is likely much higher than estimated. Fourth, we have not accounted for the potential substitution to other tobacco products.

## Conclusion

Tobacco taxation remains the single most effective public policy approach for curbing smoking rates. In this study, we confirm that the poorest will benefit the most from an increase in cigarette taxes, with more life-years gained, more premature deaths and treatment costs averted, and more cases of poverty and catastrophic health expenditures avoided compared to the richest income groups. By applying the ECEA method at the subnational level, we provide first-ever estimates of the differential impact of cigarette taxes within the Indian population. The estimates generated for each of the four states provide a powerful economic input that will equip state-level decision-makers to make a strong case for higher taxes within national debates. From this work, state-level decision makers can also make the important link between cigarette taxes and the SDGs (e.g. SDG 1: End poverty in all its forms everywhere), given the substantial impact that higher taxes will also have on alleviating poverty within the population.

## Data availability

### Underlying data

Figshare: Data inputs and data sources for impact of cigarette tax increase on health and financing outcomes in four Indian states. https://doi.org/10.6084/m9.figshare.12043074 ^16^

This project contains the following underlying data:

Extended data. xlsx
○ Table 1. Tax structure for different lengths of non-filtered and filtered cigarettes in 2018-19.
○ Table 2. Data inputs and data sources (Study data input with sources for analysis))

Data are available under the terms of the Creative Commons Attribution 4.0 International license (CC-BY 4.0).
