# Supplementary material for: Impact of cigarette tax increase on health and financing outcomes in four Indian states
Source: Gates Open Res. 2020 May 11;4:49. [Version 1] doi: 10.12688/gatesopenres.13127.1 (PMC7548764; doi:10.12688/gatesopenres.13127.1)
Supplement: Peer review history

# Peer review: Reviewer response for version 1

**DOI:** 10.21956/gatesopenres.14311.r29608

**Published:** 2020-10-09

**Authors:** Corné Van Walbeek

**Affiliations:** 1 Research Unit on the Economics of Excisable Products, School of Economics, Faculty of Commerce, University of Cape Town, Cape Town, South Africa

This is an interesting paper that considers the impact of a sizable increase in the excise tax in four Indian states. By international standards, smoking prevalence in India (and in these states) is not high. The authors show that an increase in the excise tax to 10 INR (presumably per stick, although this was not clear) plus an additional 10% tax would result in positive public health and fiscal results in all four states.

 The rationale for the choice of these four states is not clear, but one gets the impression from the Discussion that this research was done with the aim to advocate for higher taxes in these four states. That is a worthy cause, although, given the size of India, a tax at the national level would have a multiplied effect, compared to a tax increase in just four states.

 The significant contribution of this study is that it indicates that an excise tax increase is strongly progressive/pro-poor, in that the poor benefit much more than the rich.

 While the results are generally believable at the intuitive level, the authors do not provide sufficient information to allow others to replicate the study. The DOI link to the data did not work when I tested it. 

 The finding that the excise tax increase will result in many thousands of men escaping extreme poverty, requires further explanation and justification. The authors indicate (quite correctly) that the poor's demand for tobacco is more price elastic than the non-poor. Thus a greater percentage of the poor will quit when faced with a higher price of cigarettes (or tobacco more generally). But what about those smokers who do not quit smoking when they are faced with a higher price? Some may cut back on their consumption (there is evidence of that in the literature), but even for them, their total expenditure on cigarettes/tobacco may increase. For some poor smokers who are unable/unwilling to reduce their consumption, a sizable increase in the price of cigarettes will have a significant effect on their total expenditure. In some cases this will push some smokers into extreme poverty (for those who are on the verge of extreme poverty before the tax increase), or push them further into extreme poverty. The paper says nothing about this 

 The authors show that of the 2.247 million poor (lowest 20%) smokers, about 486 thousand will quit. That means that more than 1.6 million smokers do not quit and will probably increase their expenditure on cigarettes. I do not think that this is considered, and it should be.

 The authors state up front, and again in the limitations, that they use education as a proxy for income. I don't know India well, and I can accept that education and income are highly correlated, but why don't the authors simply call it what it is, namely differences in education?

 Also, the authors acknowledge that their analysis does not account for product substitution. Whether that is a big potential issue, I cannot judge. However, the authors say nothing about cross-state shopping. Is it a big potential problem? 

 I appreciate that this paper was written primarily for an Indian audience, and I suspect that  the target market would be policy makers in these four states, but the paper does not indicate whether sub-national taxes are common in India, whether other states have already implemented such tobacco taxes, and whether the constitution/government allows states to implement them. 

 As a technical document the paper is adequate and useful, but there are a number of issues that are hanging in the air, and that should, ideally, have more discussion and analysis.

Is the work clearly and accurately presented and does it cite the current literature?

Yes

If applicable, is the statistical analysis and its interpretation appropriate?

Yes

Are all the source data underlying the results available to ensure full reproducibility?

No

Is the study design appropriate and is the work technically sound?

Yes

Are the conclusions drawn adequately supported by the results?

Partly

Are sufficient details of methods and analysis provided to allow replication by others?

Partly

Reviewer Expertise:

I have worked on the economics of tobacco control for more than 20 years, focusing on taxation, tax modelling,  tobacco pricing and illicit trade, primarily in South Africa, but also in other LMICs.

I confirm that I have read this submission and believe that I have an appropriate level of expertise to confirm that it is of an acceptable scientific standard, however I have significant reservations, as outlined above.

# Peer review: Reviewer response for version 1

**DOI:** 10.21956/gatesopenres.14311.r28868

**Published:** 2020-07-07

**Authors:** Rijo M. John

**Affiliations:** 1 Centre for Public Policy Research (CPPR), Kochi, Kerala, India

**General comments:**

 This study examines the implication of an arbitrary cigarette tax increase on select outcomes such as life years gained, tax revenue generated, amount of catastrophic health expenditures averted and savings on a national health care scheme. It focuses its analysis on 4 select Indian states—Karnataka, Assam, Uttar Pradesh, and Maharashtra—chosen rather arbitrarily. This is one of the few recent studies to have examined the impact of tax increase at the subnational level and the kind of outcome variables the study focuses are quite relevant from a public health policy point of view. The study and its conclusions are quite useful for tobacco control policy in India, and taxation in particular. An important policy relevant conclusion from the study is that cigarette tax increases are disproportionately beneficial for India’s poor. This counters the age-old tobacco industry argument that tobacco taxes are regressive. This study establishes it is not.

 Although the study provides insightful analysis of the impact of a proposed tax increase, it comes with certain limitation most of which may be addressed rather quickly. Below are my specific comments on the same, not necessarily in the order of its importance.

**Specific comments:**

1. Methods para 1 says “Tax increase from the 2018-19 tax structure to INR 10 plus 10% ad valorem for all lengths of filtered cigarettes”. It is not clear why this particular combination of the tax was selected as a proposal and whether this will be in addition to the existing taxes or inclusive of the existing tax. What is also not clear is where this is proposed to be applied given that current cigarette tax structure has varied components including excise tax, national calamity contingent duty (NCCD), base GST rate (28%) and compensation cess. However, later on, from the appendix table provided, it becomes clear that the proposed tax is to be applied as a compensation cess. These details may not be obvious to a less informed reader.
2. Study population: “we used education level is a proxy for income group”. While it may be fine to use education as a proxy for income, GATS allows estimation of wealth quintiles as it provides a variety of information on the possession of different assets. Principal component analysis is usually applied to estimate such wealth quintiles from this type of surveys. The use of education as a proxy for income may be the reason for some strange prevalence figures in Table 1. For example, cigarettes, especially the filter cigarettes, are usually consumed by the relatively well-off people in India and the poor tend to smoke more of bidis. In Table 1, however, it is seen that the bottom two quintiles have cigarette smoking prevalence many times higher than the upper quintiles in UP. Although this proxy is mentioned as a limitation, I think, this is something that can be overcome using more appropriate techniques for generating income groups.
3. Cigarette price and price increase: “we obtained the 2018–19 market price of a pack of 20 cigarettes”. It is not clear why FY 2018-19 was chosen as the benchmark year for the study simulation. Union budget for FY 2019-20 re-introduced excise taxes on tobacco products, albeit nominally. This changes the tax structure and it would’ve been better had the analysis made after taking this into account. In addition, the union budget for FY 2020-21 more than doubled NCCDs applied on cigarettes. Because the benchmark year is kept as 2018-19 and there have been two rounds of tax increase including one structural change in the subsequent annual budgets, the actual estimated impact of this proposal may not be accurate as the actual prices are already higher than what is assumed in the simulation. It would be better for the simulation benchmark to reflect the actual existing taxes (both rate & structure) on cigarettes in order to estimate the true impact.
4. Price elasticity: Income group wise price elasticity estimates are available in India from a study in 2015 by Selvaraj *et al.* (2015 ^1^). The authors might want to try these out or use these at least for sensitivity analysis.
5. The subsection “Impact of tax increase to INR 10 + 10% ad valorem” need to make it clear upfront whether the proposed increase is on top of existing taxes or inclusive of existing tax. Upon examining the supplied appendix table 1, I was able to figure out that it is indeed inclusive of the existing compensation cess. But, the simulation benchmark already says the average existing ad valorem compensation cess is 16.7% while the specific cess is 3.27. The proposal is actually to increase this 3.27 to Rs.10 and decrease the 16.7% to 10%. This needs to be spelled out as such. The authors should also provide the rational for this proposal which appears to be arbitrary in nature. Why would the proposal seek a reduction in existing ad valorem rate while proposing a hike in specific tax? Why Rs. 10 specific and not 15 or 5 or 20? Why there’s an ad valorem rate at all and increase the complexity of tax structure and why not try to make a proposal that will simplify the structure instead? A discussion must address these questions.
6. Given that the model uses 2018-19 benchmark as mentioned above, it is not capturing the current excise component in existing tax structure. Moreover, to my knowledge, NCCD is applied along with the excise. The GST and ad valorem compensation cess are applied on this excise inclusive price. However, the model simulation and the estimation of taxes as given in the appendix table 1 doesn’t seem to do it this way. On what basis the proposal assumes that the proposed increase in tax to be applied on compensation cess and not on, say, excise or NCCD? Depending on where this is applied, the implications and estimated final prices and tax burden will differ. It should be also remembered that compensation cess is set to be expired 5 years from the time of GST introduction which means only remaining 2 years for its expiry. If the proposal is to apply the tax increases on compensation cess how can this be sustained when the compensation cess expires? Is proposing the increase on excise a better idea? I suggest authors discuss these issues somewhere on the paper.
7. The state-wise discussion of the results talk about the tax revenue generated from the proposed tax increase. It inadvertently give the impression that these tax revenues are accrued to the respective states implementing this tax increase. However, the compensation cess in its entirety goes to the union govt. which in turn uses this to compensate all state governments for revenue shortfalls, if any, they face up to 5 years post GST. Also, under the GST, this increase in compensation cess can’t be made unilaterally by any single state. Any proposal to increase compensation cess will be done through the GST council and shall be applicable to all states. To my knowledge, there is no mechanism for individual states to change the current tax structure on products under the GST. The authors need to clarify/discuss what their proposal will entail in practice.
8. The mentioned limitation talks about not accounting for substitution. Although there is no strong evidence of substitution between cigarettes and bidis the same cannot be said about substitution between filtered and un-filtered cigarettes. If tax is increased only on filtered cigarettes, one must realistically expect a certain amount of substitution towards unfiltered cigarettes at least by the relatively lower income group of people. It is not clear why the increase proposal cannot be made such that the increase happen simultaneously on all tiers of cigarettes. This will largely take care of such possible substitution and capture the overall impact more accurately.
9. It is also not clear how the model simulation captures the effect of income growth as there is no discussion on the same. The paper only talks about price elasticities. However, annual per capita income growth is significant in LMICs like India. While price increase has negative impact on consumption, income growth can compensate for part of these impact. A combination of price and income are important in determining true affordability of these products. It would be ideal to capture these in the model.
10. Typo: discussion second para: “prevalence in UP (11.4% vs. 6.3%)”; It should be 11.3% vs. 3.2% as given in Table 1.
11. Typo: Appendix Table 2. Data inputs and data sources (Study data input with sources for analysis)): On row 51 it says the amounts are in VND. It should be INR.
12. There have been few studies that examined the subnational impact of GST on the consumption and tax revenue of tobacco products and trends in their affordability in India. The authors might also want to look into these very recent studies as well:
  1. Guindon *et al.* (2019 ^2^).
  2. John *et al.* (2019 ^3^). 
  3. John *et al.* (2020 ^4^).
  4. Goodchild *et al. *(2020 ^5^).

Is the work clearly and accurately presented and does it cite the current literature?

Partly

If applicable, is the statistical analysis and its interpretation appropriate?

Yes

Are all the source data underlying the results available to ensure full reproducibility?

Yes

Is the study design appropriate and is the work technically sound?

Yes

Are the conclusions drawn adequately supported by the results?

Yes

Are sufficient details of methods and analysis provided to allow replication by others?

Yes

Reviewer Expertise:

I have been working on economics of tobacco control with particular interest in India for more than 15 years. I have worked extensively around issues of tobacco taxation in India and have published several peer reviewed research papers on this issue.

I confirm that I have read this submission and believe that I have an appropriate level of expertise to confirm that it is of an acceptable scientific standard, however I have significant reservations, as outlined above.

## References

[1] : Price elasticity of tobacco products among economic classes in India, 2011-2012. *BMJ Open*.2015;5(12) : 10.1136/bmjopen-2015-008180 e008180 10.1136/bmjopen-2015-008180 26656009PMC4679943

[2] : Visualizing data: Trends in smoking tobacco prices and taxes in India. *Gates Open Res*.2019;3: 10.12688/gatesopenres.12894.1 8 10.12688/gatesopenres.12894.1 30740591PMC6367560

[3] : Estimated impact of the GST on tobacco products in India. *Tob Control*.28(5) : 10.1136/tobaccocontrol-2018-054479 506-512 10.1136/tobaccocontrol-2018-054479 30219796

[4] : Trends in affordability of tobacco products before and after the transition to GST in India. *Tob Control*.2020; 10.1136/tobaccocontrol-2019-055525 10.1136/tobaccocontrol-2019-055525 32198279

[5] : Changes in the affordability of tobacco products in India during 2007/2008 to 2017/2018: a price-relative-to-income analysis. *WHO South East Asia J Public Health*.2020;9(1) : 10.4103/2224-3151.283001 73-81 10.4103/2224-3151.283001 32341226

# Peer review: Reviewer response for version 1

**DOI:** 10.21956/gatesopenres.14311.r28832

**Published:** 2020-05-26

**Authors:** M. Govinda Rao

**Affiliations:** 1 Takshashila Institution, Bengaluru, Karnataka, India

Comments on the Study, “Impact of cigarette tax increase on health and financing outcomes in four Indian states”:

 This is an important study analysing welfare implications of increases in tax on cigarettes in four Indian States namely, Karnataka, Assam, Uttar Pradesh, and Maharashtra. The authors use an extended cost-effectiveness analysis (ECEA) model developed in an earlier Disease Control Priorities Project to estimate the impact of cigarette price increases caused by tax increase in 2018-19 across five income groups in four states in India. The welfare implications are analysed in terms of smoking reduction, deaths averted due to major tobacco-attributable diseases (chronic obstructive respiratory disease (COPD), stroke, heart disease and cancer), life-years gained, treatment cost averted, number of men avoiding catastrophic health expenditures and extreme poverty, additional tax revenues raised from the tax increase, and cost savings to the Ayushman Bharat Pradhan Mantri Jan Arogya Yojana (AB-PMJAY).

 The study estimates that, in 2018-19, an increase in the price of cigarettes by 53.25% due to increase in the tax for a pack of 20 would have led to about 1.5 million men quitting smoking across the four states, with the bottom income group having 7.4 times as many quitters as the top income group. It would also have averted 665 thousand deaths due to COPD, stroke, heart disease, and cancer among current smokers due to quitting. The deaths averted due to quitting would had added an estimated 11.9 million life-years, with the bottom income group gaining 7.3 times more life-years than those in the top income group. The cost saving on hospitalisation for avoiding treatment of major tobacco-attributable diseases is estimated at Rs. 1,729 crore ($Int 955 million and the saving is estimated at 7.4 times higher for the bottom income group. This would have helped almost 75 thousand people to falling into extreme poverty. The saving on treatment cost and impoverishment is estimated at Rs. 672 crore ($Int 371 million) in AB-PMJAY and additional tax revenue generated is estimated at Rs. 4,385 crore ($Int 2,422 million).

 Thus, the study confirms that significant tax increases on filtered tobacco could improve pro-poor health outcomes and appreciable reductions in poverty. This study reaffirms that tobacco taxation is an important pro-poor public policy. This is a competent piece of work with significant public policy implications. I recommend the article for indexing.

Is the work clearly and accurately presented and does it cite the current literature?

Yes

If applicable, is the statistical analysis and its interpretation appropriate?

Yes

Are all the source data underlying the results available to ensure full reproducibility?

No

Is the study design appropriate and is the work technically sound?

Yes

Are the conclusions drawn adequately supported by the results?

Yes

Are sufficient details of methods and analysis provided to allow replication by others?

Yes

Reviewer Expertise:

Public Finance

I confirm that I have read this submission and believe that I have an appropriate level of expertise to confirm that it is of an acceptable scientific standard.
